# Supplementary material for: CG>TG mutation frequency as negative predictor of homologous recombination deficiency in ovarian and breast cancer
Source: Commun Biol. 2025 Jul 29;8:1126. doi: 10.1038/s42003-025-08529-3 (PMC12307739; doi:10.1038/s42003-025-08529-3)
Supplement: Supplementary file 1 — Supplementary Information [file 42003_2025_8529_MOESM1_ESM.pdf]

Supplemental Material

CG>TG mutation frequency as negative predictor of homologous recombination deficiency in ovarian and breast cancer

Eva Romanovsky[1], Michael Menzel[1], Klaus Kluck[1], Susanne Beck[1], Markus Ball[1], Peter Schirmacher[1,2], Daniel Kazdal[1,2], Albrecht Stenzinger[1,2,*], Jan Budczies [1,2,*]

[1]Institute of Pathology, Heidelberg University Hospital, Heidelberg, Germany.
[2]Center for Personalized Medicine (ZPM), Heidelberg, Germany

[*]Shared last authors

In this file: Suppl. Table 1-3, Suppl. Figures 1-11

Suppl. Table 1: Study cohorts and sample sizes. Class H1a* = tumors with biallelic deleterious alterations in *BRCA1* or *BRCA2*. Class H3 = tumors without alterations in the HR pathway. TCGA-OV = TCGA ovarian cancer cohort, HD-OV = in-house ovarian cancer cohort, CPTAC-OV = CPTAC ovarian cancer cohort, TCGA-BRCA = TCGA breast cancer cohort, TCGA-PANCAN = TCGA pan-cancer cohort, TCGA-PANCAN* = TCGA-PANCAN without TCGA-OV and -BRCA.

Cohort	All tumors	Class H1a* tumors	Class H3 tumors
**TCGA-OV**	411	124	126
**HD-OV**	231	19	125
**CPTAC-OV**	68	6	26
**TCGA-BRCA**	1026	79	492
**TCGA-PANCAN***	8762	110	3607
**TCGA-PANCAN* (low f_{deam})**	4928	57	2058
**TCGA-PANCAN* (high f_{deam})**	3834	53	1549
**TCGA-PANCAN**	10199	313	4225

Suppl. Table 2: Determination of cancer-specific cutpoints for the detection of HRD. Cutpoints were determined by optimization of the Youden index for separation of class H1a* and H3.

cancer type	n	n_H1aBA	n_H3	f_AUC_CI	f_p	f_cutpoint_%	HRDsum_AUC_CI	HRDsum_p	HRDsum_cutpoint	p_DeLong
**TCGA-OV**	250	124	126	0.84 (0.78-0.89)	2.50E-20	13.1	0.88 (0.83-0.92)	6.30E-24	53.5	0.18
**HD-OV**	144	19	125	0.85 (0.79-0.91)	1.10E-06	14.9	0.84 (0.76-0.91)	2.30E-06	57.5	0.81
**CPTAC-OV**	32	6	26	0.79 (0.6-0.98)	0.029	11	0.65 (0.35-0.94)	0.28	63	0.39
**TCGA-BRCA**	571	79	492	0.88 (0.85-0.92)	4.80E-28	14.9	0.92 (0.87-0.96)	5.60E-32	44.5	0.18
**TCGA-PANCAN***	3717	110	3607	0.62 (0.57-0.67)	2.00E-05	22.7	0.75 (0.7-0.81)	3.30E-19	34.5	2.80E-06
**TCGA-PANCAN* (low f)**	2115	57	2058	0.61 (0.54-0.67)	0.0067	21.5	0.76 (0.69-0.83)	5.90E-11	11.5	0.00037
**TCGA-PANCAN* (high f)**	1602	53	1549	0.7 (0.61-0.78)	1.30E-06	28.1	0.74 (0.65-0.84)	2.30E-09	36.5	0.28
**TCGA-BLCA**	93	5	88	0.95 (0.91-1)	0.00072	11.8	0.98 (0.95-1)	0.0013	65.5	0.32
**TCGA-PAAD**	116	5	111	0.85 (0.76-0.95)	0.0075	32.9	0.98 (0.95-1)	0.00031	35.5	0.044
**TCGA-STAD**	141	8	133	0.78 (0.55-1)	0.0079	15.3	0.88 (0.78-0.97)	0.00037	36.5	0.32
**TCGA-UCEC**	93	17	76	0.68 (0.49-0.87)	0.02	18.4	0.66 (0.44-0.87)	0.048	56.5	0.84
**TCGA-SARC**	125	7	118	0.74 (0.58-0.91)	0.031	15.5	0.78 (0.64-0.93)	0.02	38.5	0.94
**TCGA-COAD**	143	3	140	0.84 (0.67-1)	0.048	38.6	0.34 (0-0.99)	0.34	62.5	0.047
**TCGA-GBM**	146	3	143	0.78 (0.63-0.93)	0.1	36.3	0.71 (0.51-0.9)	0.22	9.5	0.63
**TCGA-LIHC**	137	3	134	0.77 (0.6-0.94)	0.11	8.9	0.6 (0.2-0.99)	0.58	15.5	0.45
**TCGA-LUSC**	100	11	89	0.64 (0.45-0.83)	0.13	5.2	0.75 (0.57-0.92)	0.0085	47.5	0.37
**TCGA-HNSC**	169	5	164	0.66 (0.4-0.92)	0.23	16	0.75 (0.52-0.98)	0.055	23.5	0.52
**TCGA-PRAD**	273	4	269	0.65 (0.23-1)	0.29	21.4	0.94 (0.86-1)	0.0026	20.5	0.13
**TCGA-TGCT**	106	15	91	0.42 (0.3-0.54)	0.32	24.5	0.45 (0.3-0.59)	0.52	12.5	0.69
**TCGA-SKCM**	68	3	65	0.46 (0.33-0.59)	0.81	17.3	0.68 (0.22-1)	0.3	34.5	0.39
**TCGA-THCA**	414	5	409	0.52 (0.2-0.85)	0.86	1.7	0.4 (0.24-0.56)	0.35	NA	0.55
**TCGA-CESC**	87	6	81	0.51 (0.19-0.84)	0.93	15.1	0.64 (0.34-0.93)	0.27	26.5	0.31

Suppl. Table 3: Frequency of CG>TG mutations (f_{deam}) for each SBS. Sorted by highest to lowest frequency.

SBS	definition	f_{deam}
**SBS1**	Spontaneous deamination of 5-methylcytosine, clock-like signature	0.8892
**SBS6**	Defective DNA mismatch repair	0.4723
**SBS10b**	Polymerase epsilon exonuclease domain mutations	0.4537
**SBS15**	Defective DNA mismatch repair	0.3976
**SBS87**	Thiopurine chemotherapy treatment	0.3859
**SBS98**	Unknown	0.1374
**SBS7a**	Ultraviolet light exposure	0.0809
**SBS7b**	Ultraviolet light exposure	0.0802
**SBS44**	Defective DNA mismatch repair	0.0794
**SBS29**	Tobacco chewing	0.0681
**SBS32**	Azathioprine treatment	0.068
**SBS94**	Unknown	0.0639
**SBS97**	Unknown	0.0575
**SBS25**	Chemotherapy treatment	0.0559
**SBS18**	Damage by reactive oxygen species	0.0556
**SBS20**	Concurrent POLD1 mutations and defective DNA mismatch repair	0.053
**SBS5**	Unknown, clock-like signature	0.0519
**SBS86**	Unknown chemotherapy treatment	0.0517
**SBS30**	Defective DNA base excision repair due to NTHL1 mutations	0.0511
**SBS2**	Activity of APOBEC family of cytidine deaminases	0.047
**SBS93**	Unknown	0.0436
**SBS14**	Concurrent polymerase epsilon mutation and defective DNA mismatch repair	0.0435
**SBS10c**	Defective POLD1 proofreading	0.0403
**SBS23**	Unknown	0.0381
**SBS96**	Unknown	0.0372
**SBS40a**	Unknown	0.0361
**SBS41**	Unknown	0.0356
**SBS24**	Aflatoxin exposure	0.035
**SBS92**	Tobacco smoking	0.0301
**SBS88**	Colibactin exposure (E.coli bacteria carrying pks pathogenicity island)	0.0283
**SBS40c**	Unknown	0.028
**SBS34**	Unknown	0.0266
**SBS31**	Platinum chemotherapy treatment	0.0262

**SBS19**	Unknown	0.0248
**SBS39**	Unknown	0.0239
**SBS10a**	Polymerase epsilon exonuclease domain mutations	0.0233
**SBS33**	Unknown	0.0227
**SBS36**	Defective DNA base excision repair due to MUTYH mutations	0.022
**SBS9**	Polymerase eta somatic hypermutation activity	0.0189
**SBS26**	Defective DNA mismatch repair	0.018
**SBS37**	Unknown	0.0174
**SBS38**	Indirect effect of ultraviolet light	0.0166
**SBS21**	Defective DNA mismatch repair	0.014
**SBS40b**	Unknown	0.0135
**SBS89**	Unknown	0.0128
**SBS84**	Activity of activation-induced cytidine deaminase (AID)	0.0118
**SBS28**	Unknown	0.0116
**SBS11**	Temozolomide treatment	0.0103
**SBS7c**	Ultraviolet light exposure	0.0099
**SBS22b**	Aristolochic acid exposure	0.0092
**SBS10d**	Defective POLD1 proofreading	0.0087
**SBS16**	Unknown	0.0081
**SBS42**	Haloalkane exposure	0.0073
**SBS91**	Unknown	0.0067
**SBS8**	Unknown	0.0067
**SBS35**	Platinum chemotherapy treatment	0.0065
**SBS3**	Defective homologous recombination DNA damage repair	0.0063
**SBS7d**	Ultraviolet light exposure	0.0058
**SBS4**	Tobacco smoking	0.0049
**SBS17a**	Unknown	0.0037
**SBS13**	Activity of APOBEC family of cytidine deaminases	0.0037
**SBS22a**	Aristolochic acid exposure	0.0019
**SBS17b**	Unknown	0.0017
**SBS90**	Duocarmycin exposure	0.0008
**SBS85**	Indirect effects of activation-induced cytidine deaminase (AID)	0.0007
**SBS99**	Melphalan exposure	0.0004
**SBS12**	Unknown	0.0003

Suppl. Figure 1

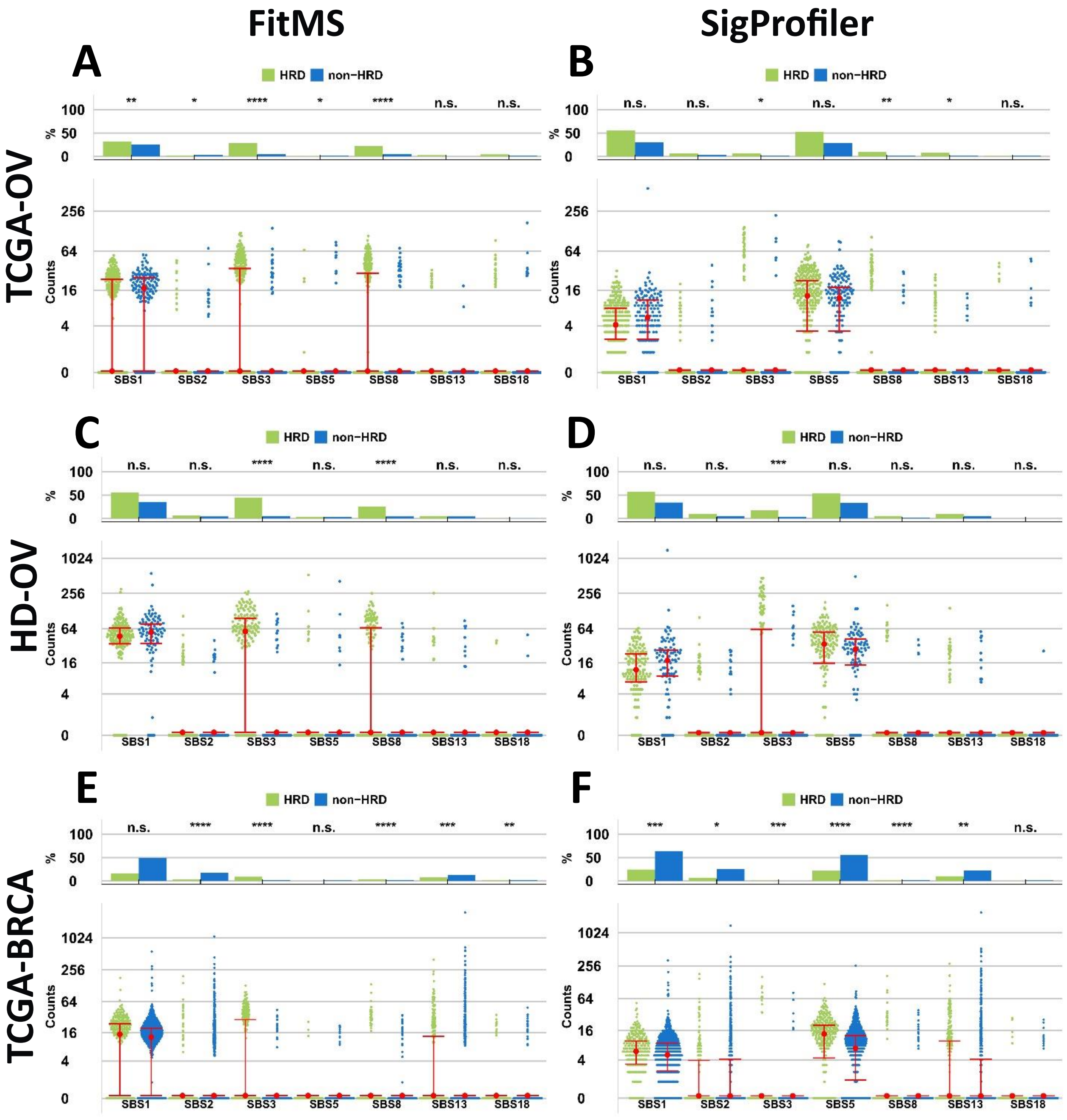

Suppl. Figure 1: Correlation of the presence and the level of SBS mutational signatures with HRD status. Analysis of SBS1, SBS2, SBS3, SBS5, SBS8, SBS13, and SBS18 detected by the bioinformatic algorithms FitMS and SigProfiler for the ability to separate between HR-deficient (class H1a*) and HR-proficient (class H3) tumors. **A-B** TCGA-OV**, C-D** HD-OV, and **E-F** TCGA-BRCA.

Suppl. Figure 2

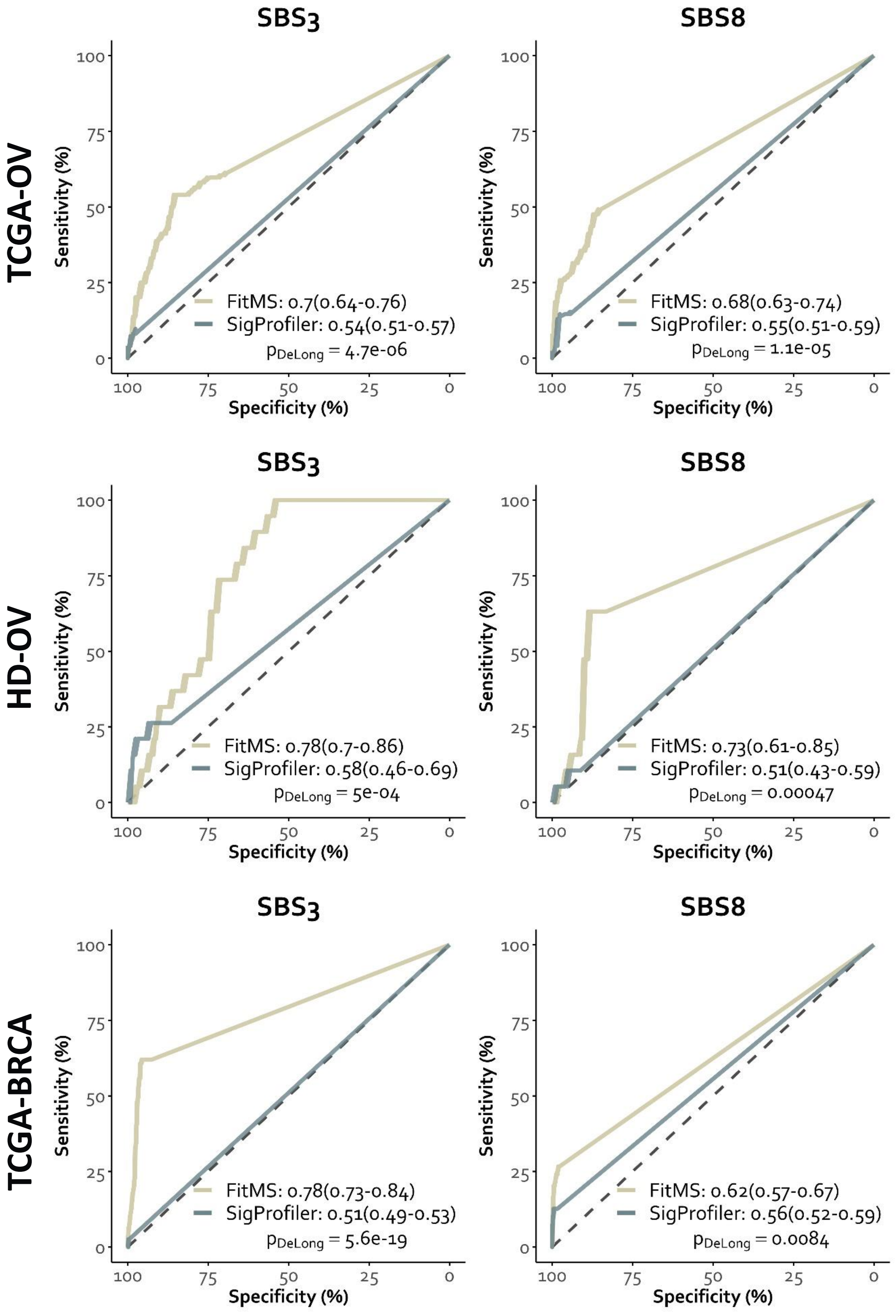

Suppl. Figure 2: Evaluation of alternative methods for the fitting of mutational signatures. FitMS with was run with ovarian cancer as prespecified cancer type. SigProfiler was run without exclusion of signatures putatively caused by sequencing artefacts.

Suppl. Figure 3

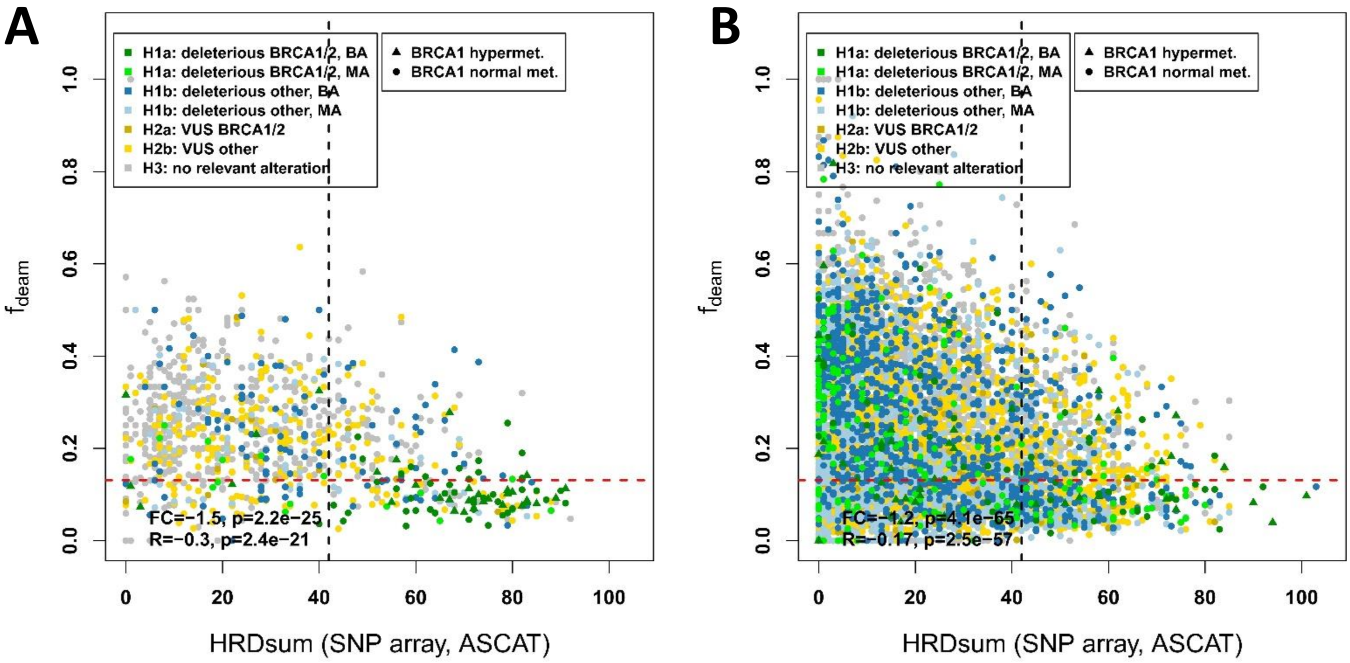

Suppl. Figure 3: Correlation analysis of f_{deam} and HRDsum in A breast cancer (TCGA-BRCA) and **B** pan-cancer w/o ovarian and breast cancer (TCGA-PANCAN*).

Suppl. Figure 4

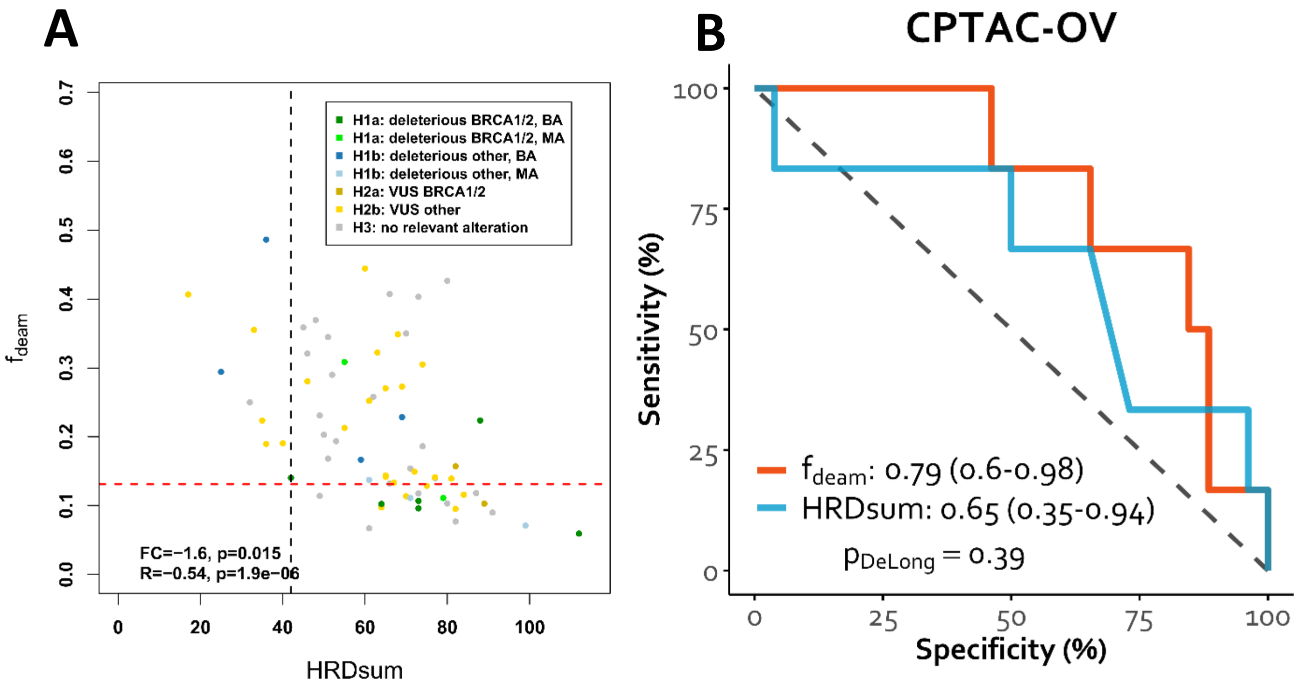

Suppl. Figure 4: Validation of f_{deam} as biomarker for the detection of HRD in CPTAC-OV (n=68). A Correlation analysis of f_{deam} and HRDsum. **B** Separation between HR-deficient and -proficient tumors by f_{deam} and HRDsum.

Suppl. Figure 5

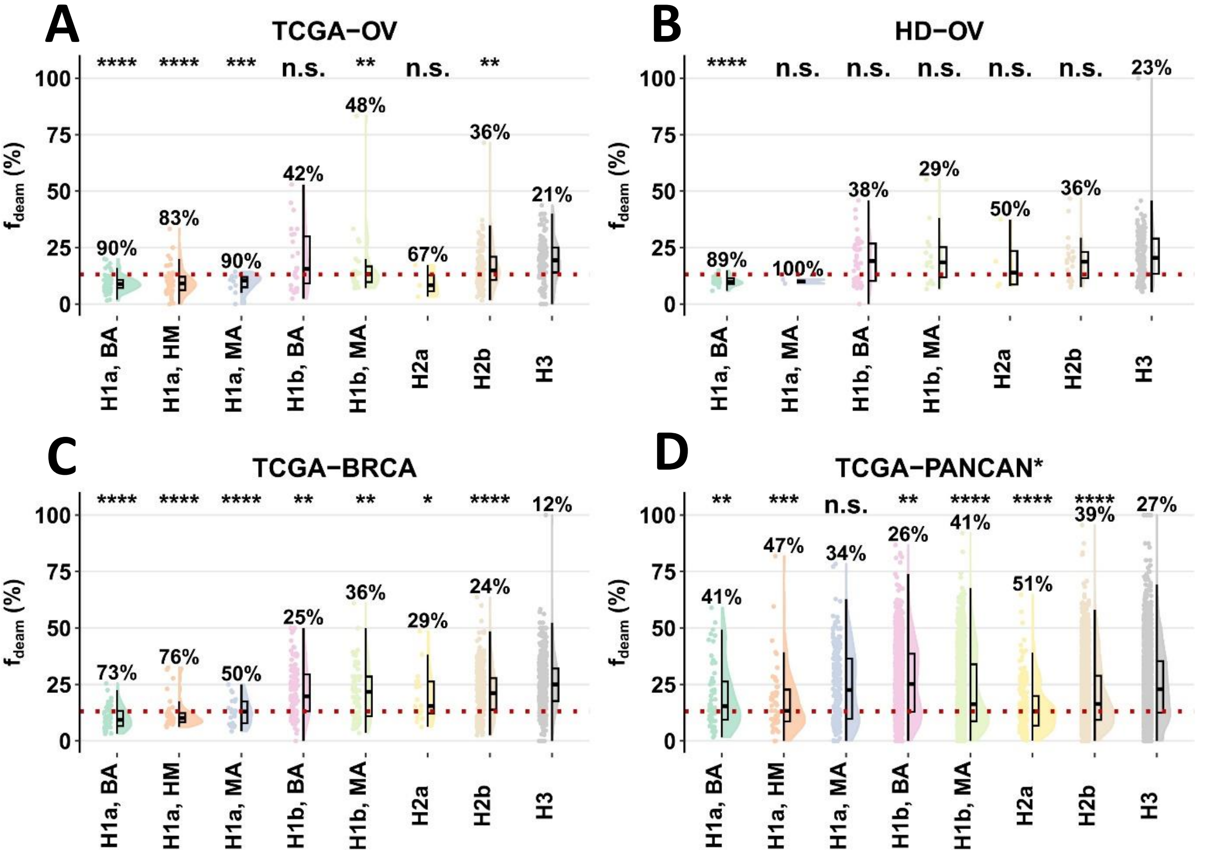

Suppl. Figure 5: Association of the levels of f_{deam} with tumor classification by alterations in *BRCA1*, *BRCA2*, and other genes in the HR pathway. H1a = deleterious alteration in *BRCA1/2*, BM = biallelic, HM = *BRCA1* hypermethylation, MA = monoallelic, H1b = deleterious alteration in other genes of the HR pathway, H1a = VUS in *BRCA1/2*, H1b = VUS in other genes of the HR pathway, and H3 = not in classes H1a, H1b, H2a, and H2b. Percentages refer to the numbers of tumors below the cutpoint f_{deam} = 13.1%.

Suppl. Figure 6

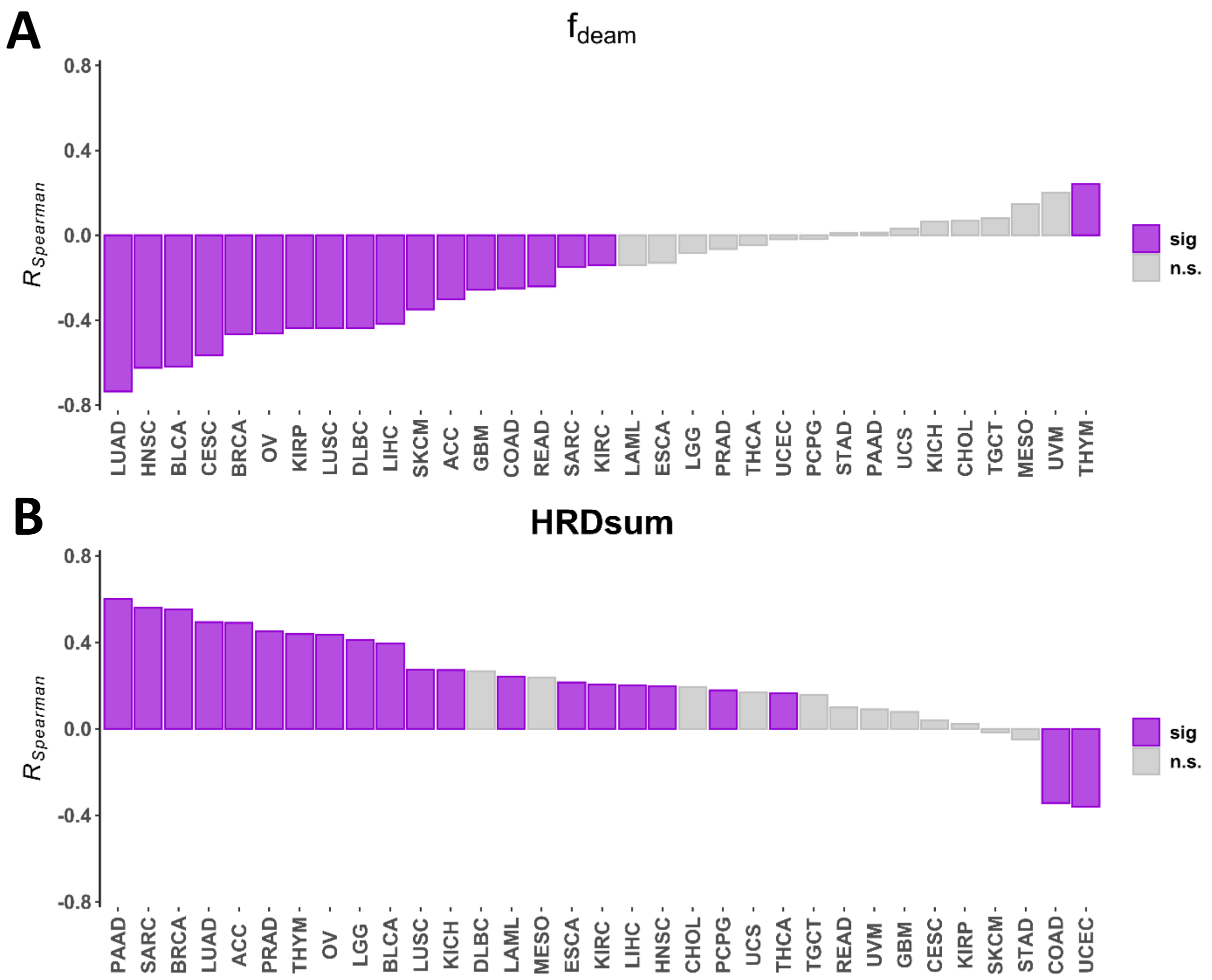

Suppl. Figure 6: Correlation of studied HRD markers with TMB across cancer types. A Correlation of f_{deam} and TMB. **B** Correlation of HRDsum and TMB. Stratified analysis across all TCGA cancer types, sorted by Spearman correlation coefficient. Colored bars indicate statistically significant correlations after multiple testing correction (FDR=5%). TMB = number of SBSs in the genomic region covered by WES.

Suppl. Figure 7

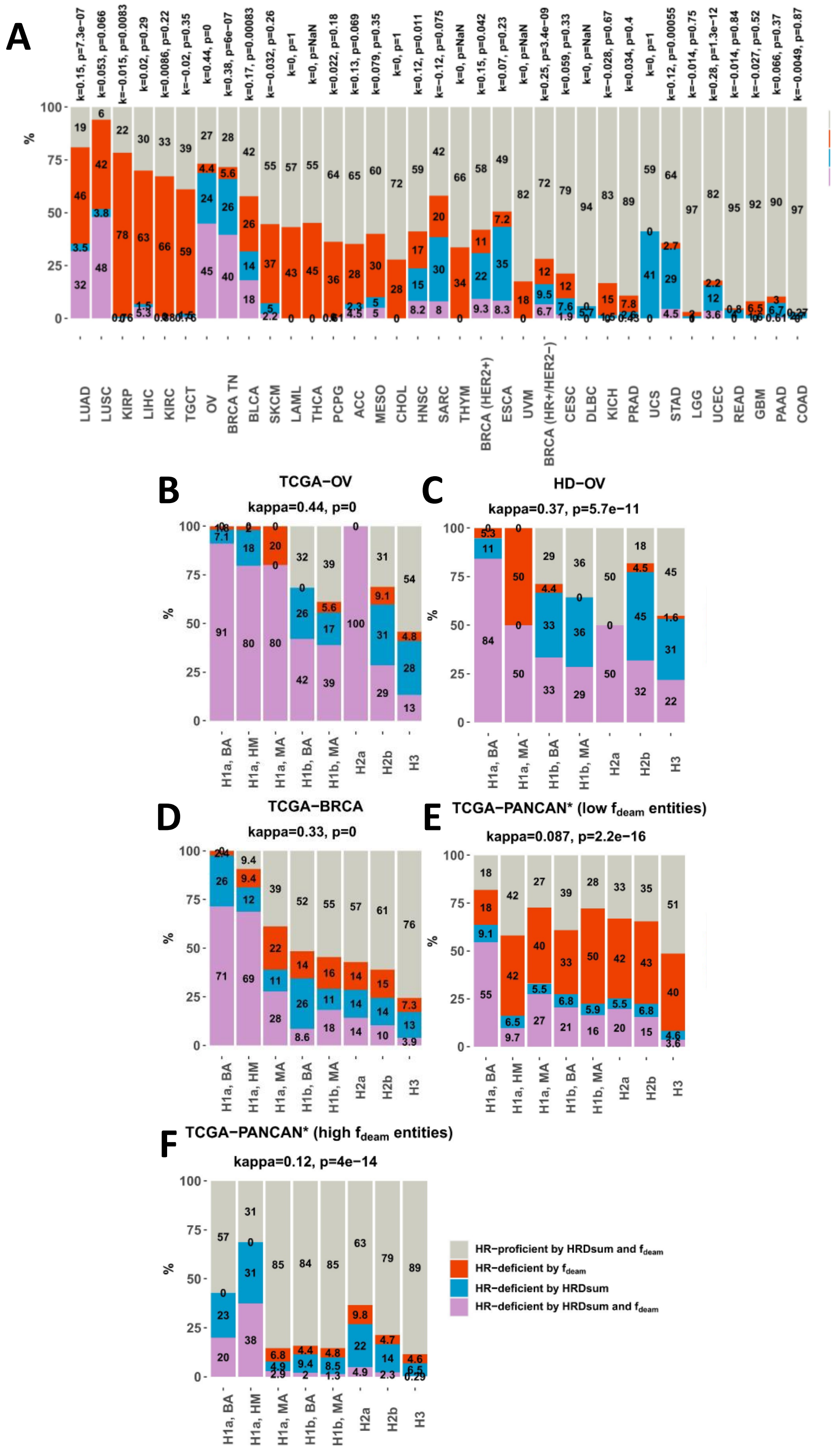

Suppl. Figure 7: Comparison of HRD detection using f_{deam} (cutpoint: 13.1%) and HRDsum (cutpoint: 42). A Analysis of the agreement of the two biomarkers in 35 cancer types. **B-F** In-depth analysis of tumors with specific mutations in the HR pathway.

Suppl. Figure 8

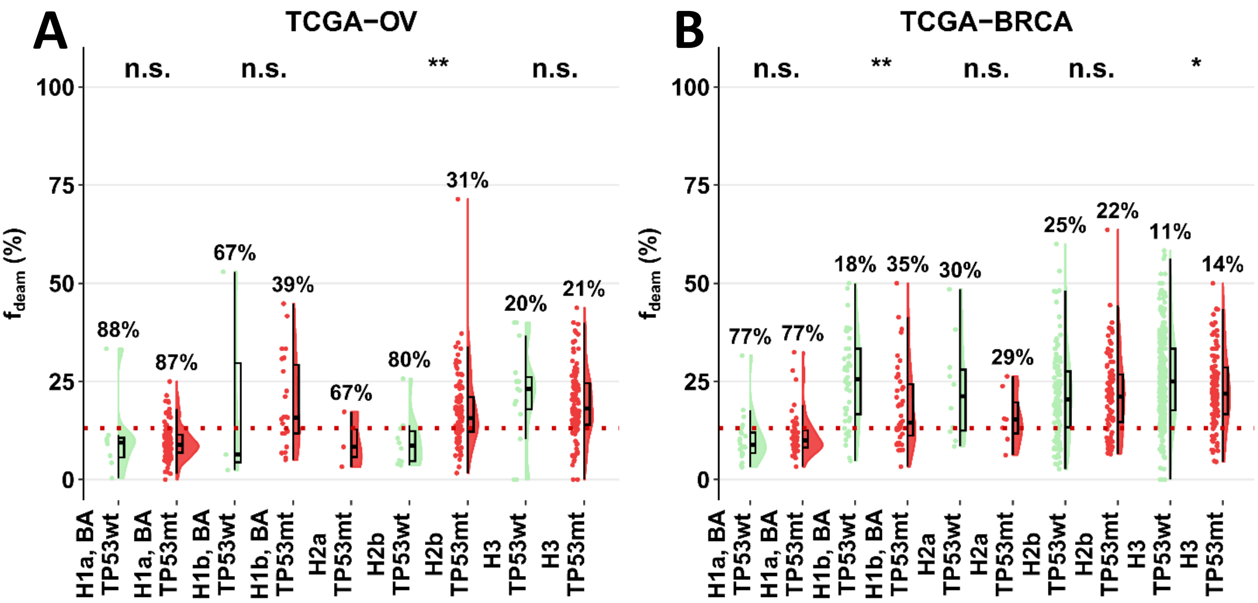

Suppl. Figure 8: Association of the levels of f_{deam} with tumor classification by alterations in *TP53* in the HR pathway. TP53mt = tumors with *TP53* mutations, and TP53wt = tumors with *TP53* wildtypes.

Suppl. Figure 9

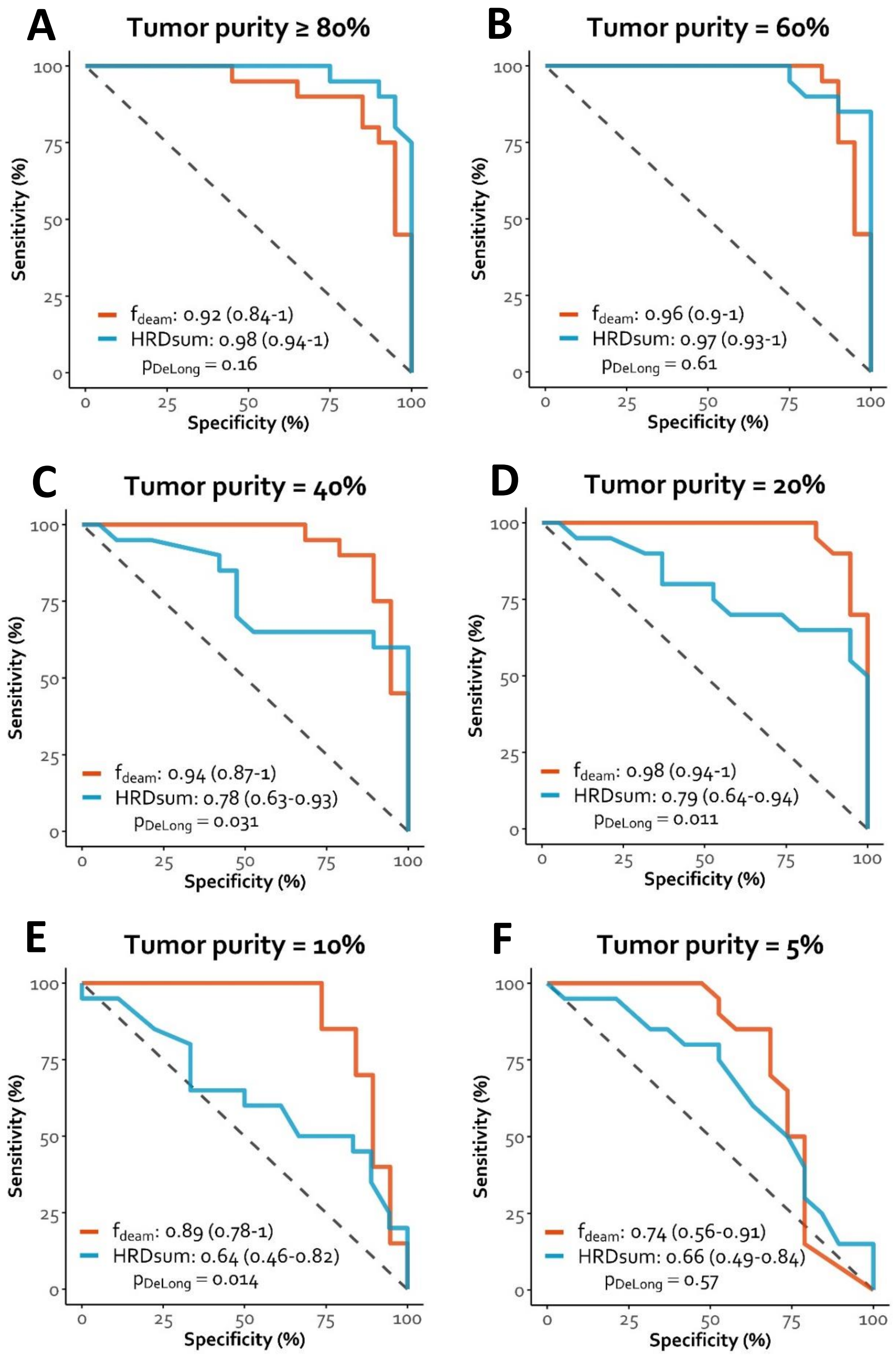

Suppl. Figure 9: Low tumor purity as confounder for HRD detection. Starting from a cohort of ovarian cancers with tumor purity ≥ 80% including 20 HR-deficient (class H1a*) and 20 HR-proficient (class H3) tumors, we simulated cohorts with lower tumor purity of 60%, 40%, 20%, 10%, and 5%. The new biomarker f_{deam} significantly outperformed HRDsum for tumor purity 40%, 20%, and 10%.

Suppl. Figure 10

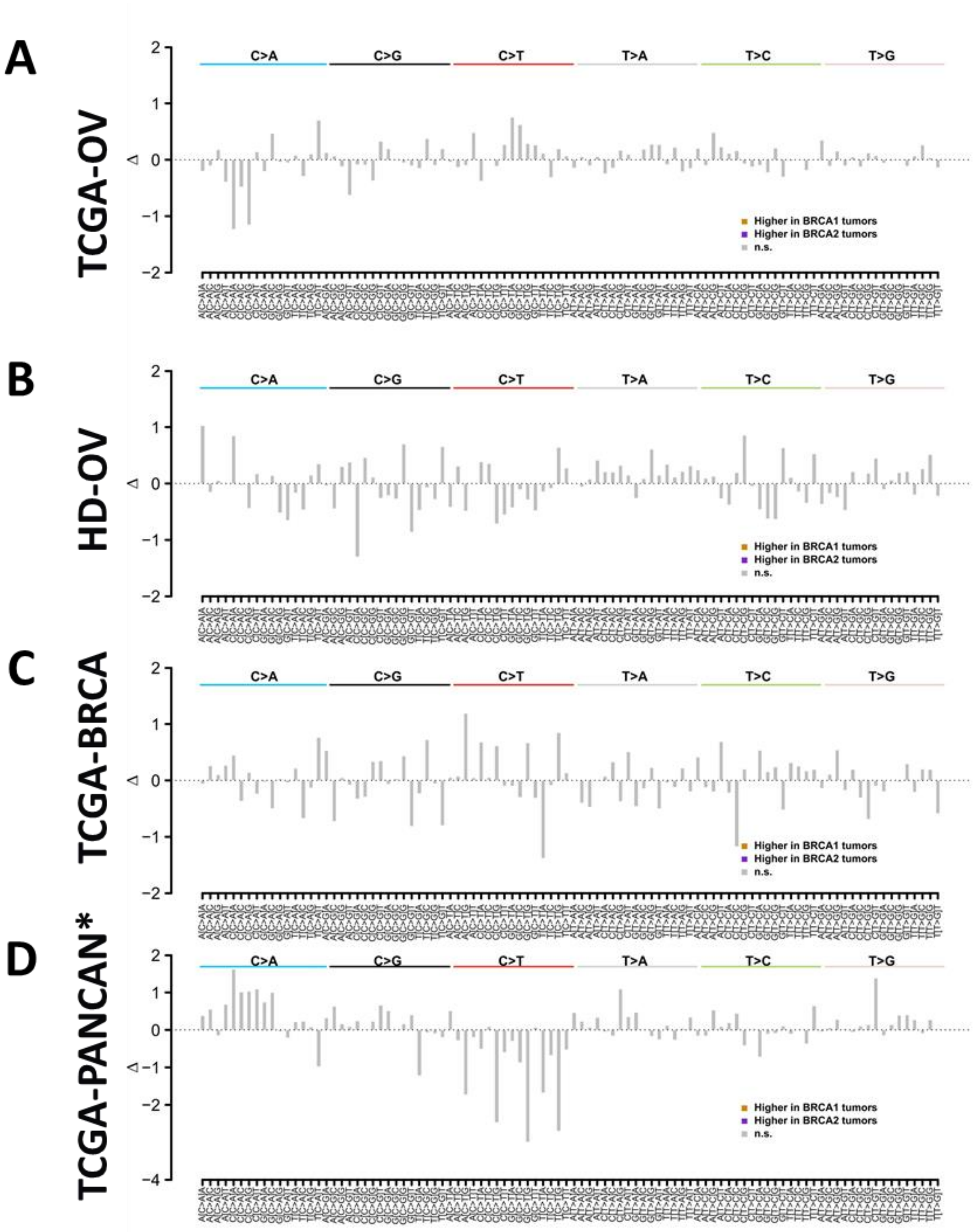

Suppl. Figure 10: Comparison of the mutation profiles between *BRCA1*- und *BRCA2*-mutated tumors. A TCGA-OV **B** HD-OV **C** TCGA-BRCA, and **D** TCGA-PANCAN*.

Suppl. Figure 11

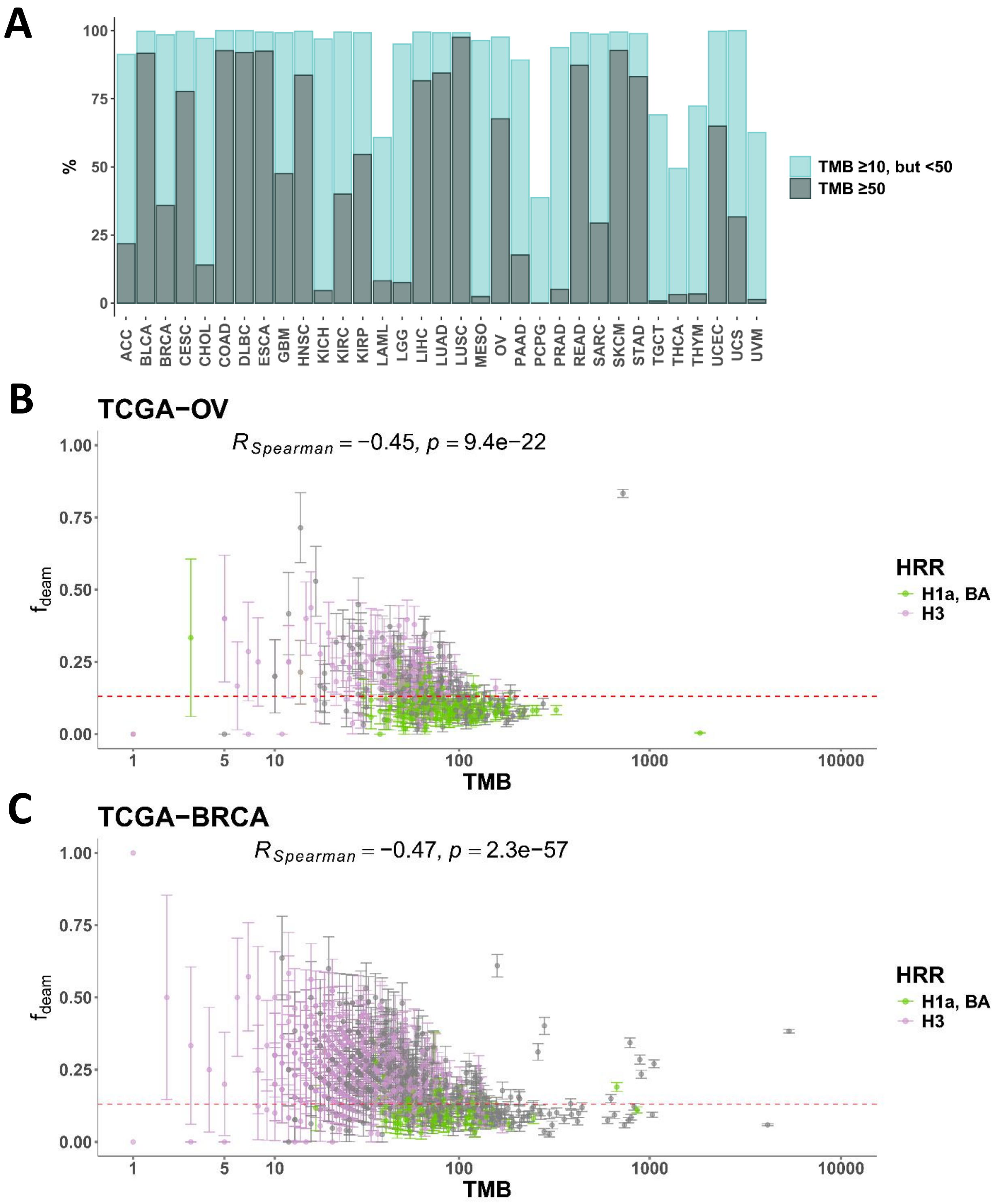

Suppl. Figure 11: Low TMB as confounder of f_{deam}. A Percentage of tumors with TMB of at least 10 (and least 50) mutations. **B/C** Scatterplot of f_{deam} (including error bars showing the standard deviation) and TMB. TMB = number of SBSs in the genomic region covered by WES.
